# Supplementary material for: The interactome of intact mitochondria by cross-linking mass spectrometry provides evidence for coexisting respiratory supercomplexes
Source: Mol Cell Proteomics. 2017 Dec 8;17(2):216–32. doi: 10.1074/mcp.RA117.000470 (PMC5795388; doi:10.1074/mcp.RA117.000470)
Supplement: Supplemental Data [file supp_RA117.000470_133922_0_supp_23978_fzffwf.zip › spectra_annotation/mito_DR_spectra_annotation/439-1-7-1-7-1.pdf]

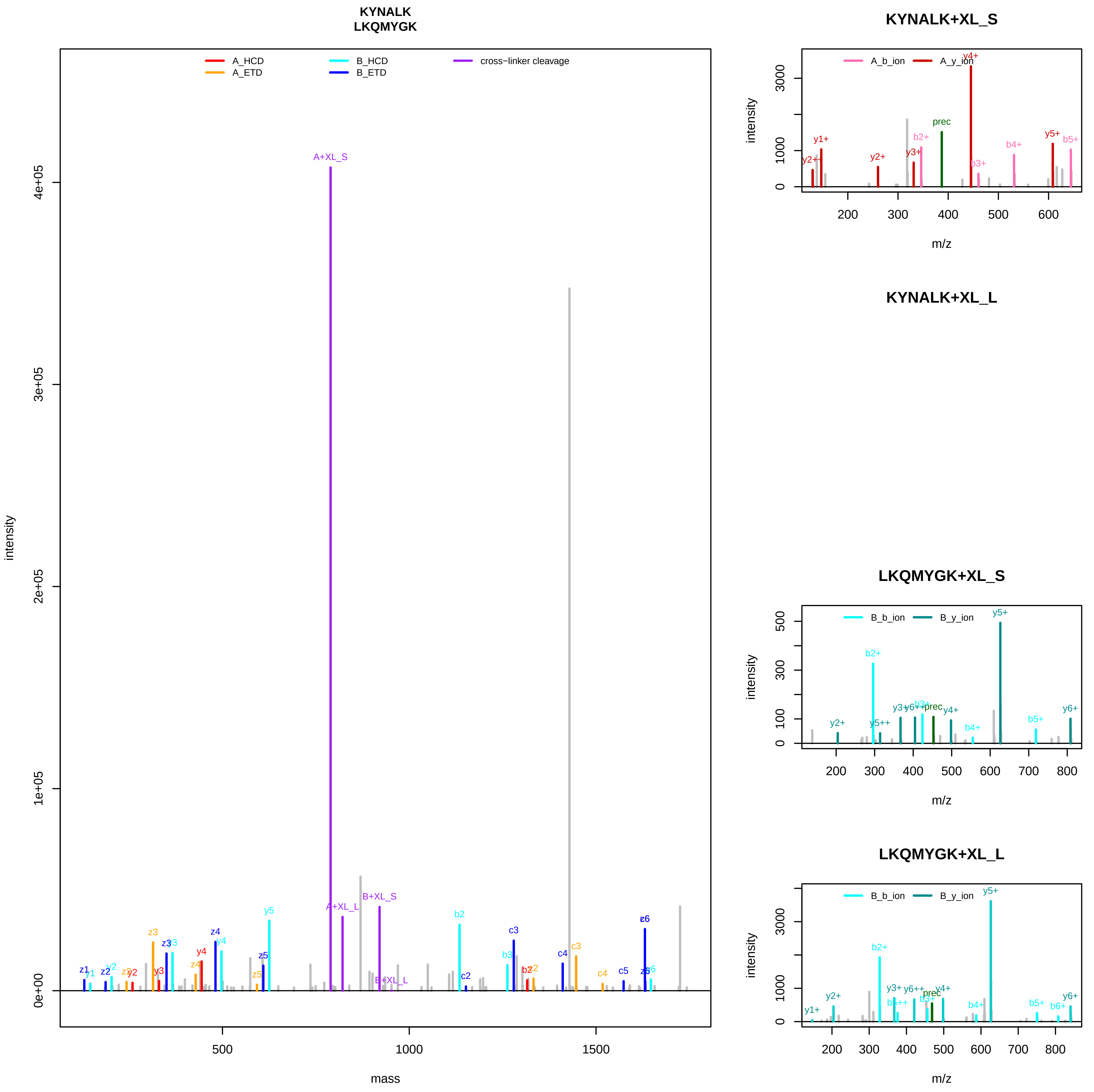

KYNALK
LKQMYGK
A_HCD
A_ETD
B_HCD
B_ETD
cross−linker cleavage
A+XL_S
A+XL_L
B+XL_S
B+XL_L
z1 y1 z2 y2 z2 y2 y3 z3 z3 y3 z4 y4 y4 z4 z5 y5 z5
b2 c2 b3 c3 b2 z2 c3 c4 c4 c5 z6 y6 c6
intensity
0e+00
1e+05
2e+05
3e+05
4e+05
500
1000
1500
mass
KYNALK+XL_S
A_b_ion
A_y_ion
y2+ y1+ y2+ y3+ b2+ prec b3+ y4+ b4+ y5+ b5+
intensity
m/z
KYNALK+XL_L
LKQMYGK+XL_S
B_b_ion
B_y_ion
y2+ b2+ y5++ y3+ y6++ b3+ prec y4+ b4+ y5+ b5+ y6+
intensity
m/z
LKQMYGK+XL_L
B_b_ion
B_y_ion
y1+ y2+ b2+ y3+ b3++ y6++ b3+ prec y4+ b4+ y5+ b5+ b6+ y6+
intensity
m/z
